# Multisensory Stimulation Can Induce an Illusion of Larger Belly Size in Immersive Virtual Reality

**DOI:** 10.1371/journal.pone.0016128

**Published:** 2011-01-19

**Authors:** Jean-Marie Normand, Elias Giannopoulos, Bernhard Spanlang, Mel Slater

**Affiliations:** 1 EVENT Lab, Facultat de Psicologia, Universitat de Barcelona, Barcelona, Spain; 2 Institució Catalana Recerca i Estudis Avançats (ICREA), Barcelona, Spain; 3 Department of Computer Science, University College London, London, United Kingdom; CNRS - University Paul Sabatier, Toulouse, France

## Abstract

**Background:**

Body change illusions have been of great interest in recent years for the understanding of how the brain represents the body. Appropriate multisensory stimulation can induce an illusion of ownership over a rubber or virtual arm, simple types of out-of-the-body experiences, and even ownership with respect to an alternate whole body. Here we use immersive virtual reality to investigate whether the illusion of a dramatic increase in belly size can be induced in males through (a) first person perspective position (b) synchronous visual-motor correlation between real and virtual arm movements, and (c) self-induced synchronous visual-tactile stimulation in the stomach area.

**Methodology:**

Twenty two participants entered into a virtual reality (VR) delivered through a stereo head-tracked wide field-of-view head-mounted display. They saw from a first person perspective a virtual body substituting their own that had an inflated belly. For four minutes they repeatedly prodded their real belly with a rod that had a virtual counterpart that they saw in the VR. There was a synchronous condition where their prodding movements were synchronous with what they felt and saw and an asynchronous condition where this was not the case. The experiment was repeated twice for each participant in counter-balanced order. Responses were measured by questionnaire, and also a comparison of before and after self-estimates of belly size produced by direct visual manipulation of the virtual body seen from the first person perspective.

**Conclusions:**

The results show that first person perspective of a virtual body that substitutes for the own body in virtual reality, together with synchronous multisensory stimulation can temporarily produce changes in body representation towards the larger belly size. This was demonstrated by (a) questionnaire results, (b) the difference between the self-estimated belly size, judged from a first person perspective, after and before the experimental manipulation, and (c) significant positive correlations between these two measures. We discuss this result in the general context of body ownership illusions, and suggest applications including treatment for body size distortion illnesses.

## Introduction

In this paper we show how it is possible to induce a body distortion illusion in immersive virtual reality based on first person perspective of a virtual body that receives synchronous visual-tactile and synchronous visual-motor correlation. The illusion induced is that the participant is significantly fatter than he really is, demonstrated by subjective evidence using a questionnaire, and additionally through direct estimates of body size from a first person perspective before and after the experiment. Research over the past decade in cognitive neuroscience has demonstrated that it is quite straightforward to experimentally induce the illusion in people that their bodies have suddenly changed in various ways. Botvinick and Cohen [1] showed that it is possible to induce an illusion of ownership of a rubber arm, even though the rubber arm does not look like the person's own arm. The rubber arm is placed in a plausible position on a table, and the corresponding real one is hidden behind a screen and approximately parallel with the rubber hand. The experimenter simultaneously taps and/or strokes the real and rubber hand so that the subject sees the rubber hand being stimulated and feels the corresponding stimulation on the real hand. When the visual and tactile stimulation are synchronous, and the stimulation is applied in the same place on the rubber hand as it is felt on the real hand, then 80% of people will feel within about 15s of stimulation that the rubber hand is their hand [2], provided that the rubber hand and real hand are close to one another (15–18cm). When the visual-tactile stimulation is asynchronous the illusion does not occur or occurs to a much lesser extent.

The same fundamental principle, of synchronous multisensory stimulation has been applied to produce whole body illusions. Lenggenhager et al. [3] and Ehrsson [4] used head-mounted displays to stream video images to subjects from cameras behind them, so that through the HMD the subject saw a live video of themselves from behind. Subjects had the illusion of being drawn forward towards the body representation that they saw in front of them in the Lenggenhager et al. experiment, as a result of experiencing synchronous visual-tactile stimulation on the back of the body seen in front of them, but felt on their own back. In the Ehrsson setup subjects had the experience that the video image that they saw in front of themselves was an ‘empty shell’ and that they felt themselves to be located behind where their real body was located. This was achieved through visual-tactile stimulation on the chest of the subject and simultaneously underneath the video cameras behind the subject. The subject would apparently therefore see the visual tapping in the virtual location of their chest, while feeling it on their actual chest. In both cases asynchronous visual-tactile stimulation did not result in the out-of-body illusion. An experiment comparing the results of these two experimental paradigms has been presented in [5].

Petkova and Ehrsson [6] showed how to induce the illusion of body ownership of a manikin seen to replace the subject's body from a first person perspective. Subjects wore head-mounted displays linked to a pair of cameras mounted on the head of a manikin, so that when the subject looked through the HMD they would see the manikin's body instead of their own. Synchronous visual-tactile stimulation on the middle front of the subject's body, and seen from a first person perspective on the manikin's body, induced an illusion that the person's body had become that of the manikin. Asynchronous stimulation did not result in the illusion.

There are typically three types of evidence used to indicate the illusion. The first is subjective, based on a questionnaire usually modified from an original one presented in [1]. There are normally a number of questions that indicate the illusion and others that are thought of as control questions that are apparently similar but do not indicate the illusion. These questions are scored on a Likert scale. The second type of measurement, also introduced in [1] is termed ‘proprioceptive drift’. In the case of the rubber hand illusion (RHI), for example, subjects are asked to blindly point to the position of their real hand before the experiment, and then again immediately after. A drift towards the position of the rubber hand indicates that the brain has recalibrated peripersonal space, so that the position of the hand has been recalculated based on the position of the rubber hand. A version of this was used in [3] where subjects had to walk blindly to where they felt themselves to have been both before and after the experiment, and in the synchronous condition they tended to walk closer to the image of the body that they had seen in front. A third measure is one based on response to threat. This was introduced by Armel and Ramachandran [7] in the context of the RHI - a threat to the rubber hand in the synchronous condition caused a skin conductance response (arousal in response to the expectation of pain) but this did not occur in the asynchronous condition. This approach was also used in [4], [6], [8]. Of particular importance are correlations between the subjective (questionnaire based) results and the more objective results - such as the drift measurements or physiological responses. Such correlations between data generated in quite different ways reinforce the occurrence of the various illusions.

These types of illusions have been shown to operate well in virtual reality. Slater et al. [9] showed that a virtual arm projected on a stereo powerwall (including head-tracking) and seen as virtually projecting out of the subject's real body produced ownership over the virtual limb akin to the RHI. In this case a tracked Wand was used to tap the hand of the subject, and the Wand was represented as a virtual ball synchronously or asynchronously tapping the corresponding virtual hand. This used the Botvinick and Cohen questionnaire, proprioceptive drift, and a measure of arm movement in response to movement of the virtual arm as measured by electromyogram. Sanchez-Vives et al. [10] showed that the illusion could also be produced by synchronous visual-motor actions - when the virtual hand moved synchronously with the real hand, implemented using a data glove, but not when the movement was asynchronous. A similar study by Yuan and Steed [11] also showed a skin conductance response to a threat to the virtual hand but not when the virtual hand was replaced by an abstract cursor. Slater et al. [12] showed that a whole body ownership illusion can be induced in men that their body has transformed to being female. This was achieved using first person perspective and synchronous visual-tactile stimulation, as displayed through a wide field-of-view HMD. Gonzalez-Franco et al. [13] showed that a whole body ownership illusion with respect to an avatar reflected in a virtual mirror could be induced by synchronous upper body movements.

The illusions of limb or body replacement described above rely on conflicting multisensory stimulation that is resolved in favor of visual dominance. The hand or body that is seen to be tapped, while the taps are felt synchronously, is the one to which ownership is attributed. There is another class of body distortion illusions that operate by inducing a contradiction between (self-touch) tactile and proprioceptive sensations. When vibrations are applied, for example, to the muscle spindles around the elbow, and the eyes of the subject are closed, it is possible to induce the illusion that the forearm is opening (or closing - depending on exactly where the vibration is applied) [14]. Now suppose that while the subject has the sensation that their forearm is moving outwards they are touching the tip of their nose with the fingers of the apparently moving arm, the brain has a contradiction to resolve (the hand is moving away from the head but there is continuous touching of the nose). It typically resolves this contradiction by generating the illusion that the nose is extending in length, the so-called Pinocchio Illusion. Similarly, if the subject is touching both sides of the waist with his hands, and both elbows are stimulated to generate the illusion that the forearms are opening, this induces the illusion that the body is widening, becoming fatter [15], [16].

In the present experiment participants see, from a first person perspective, a larger virtual body substituting their own. This together with self-induced synchronous visual-tactile stimulation and visual-motor correlation, seen on the virtual body and felt on the real body in the corresponding location was sufficient to generate an ownership illusion and a feeling that the belly had increased in size.

## Results

### Experimental Design

Participants entered an immersive virtual reality through a stereoscopic wide field-of-view head-tracked head-mounted display (HMD) and saw themselves with a significantly fatter body than they really had. They self-applied synchronous or asynchronous visual-tactile stimulation to their stomach area by means of a rod that could poke their belly, and a corresponding virtual rod that was seen to touch their virtual belly. Our hypothesis was that the synchronous stimulation would be more likely to result in a body size distortion illusion than the asynchronous.

**Figure 1:**
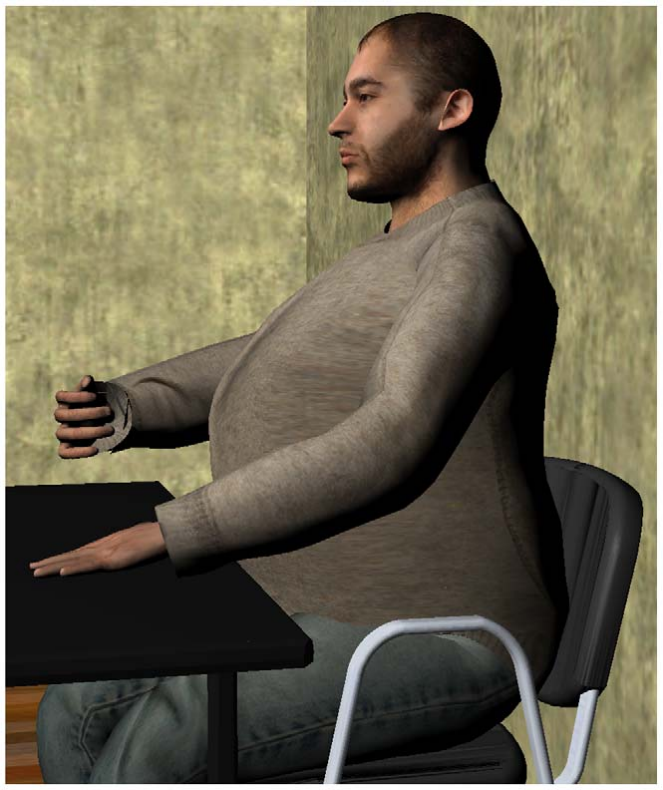
An avatar with large belly size representing the participant.

This was a within-groups experimental design where each participant repeated the experiment twice in counterbalanced order, one time with synchronous and the other time with asynchronous stimulation. Although 24 participants were originally recruited for the experiment there were two incomplete data records, and the analysis was carried out on the remaining 22 participants. This group had mean age 26±5 (S.D.) years. There were 12 who first experienced the synchronous condition and 10 who first experienced the asynchronous condition. The time between their two exposures was about 5 minutes taking into account removal of the HMD, completing a questionnaire and short interview, and putting on the HMD again for the second exposure. The virtual character with an enlarged belly is shown in Figure 1, and an overview of the experiment can be seen in online supporting information (Movie S1).

**Figure 2:**
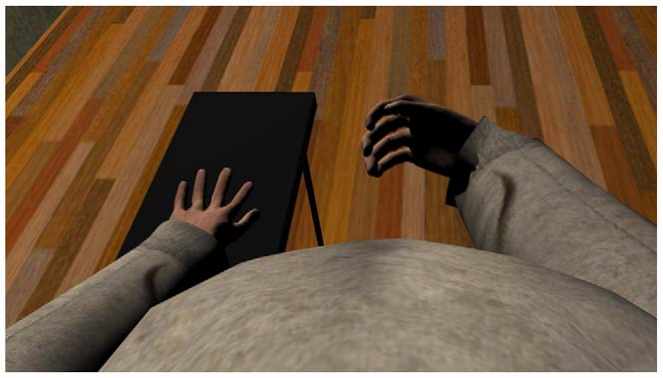
First person perspective of the virtual body with an inflated belly.

Upon arrival and after reading an information sheet describing the procedures of the experiment, and reading and signing an informed consent form, they were seated at a table and put on a head-mounted display. The scenario that they then saw consisted of a virtual room including a virtual table registered with the real table at which they sat. They were told to look down and would see a virtual body from first person perspective, i.e., where their real body should normally be seen (Figure 2).

**Figure 3:**
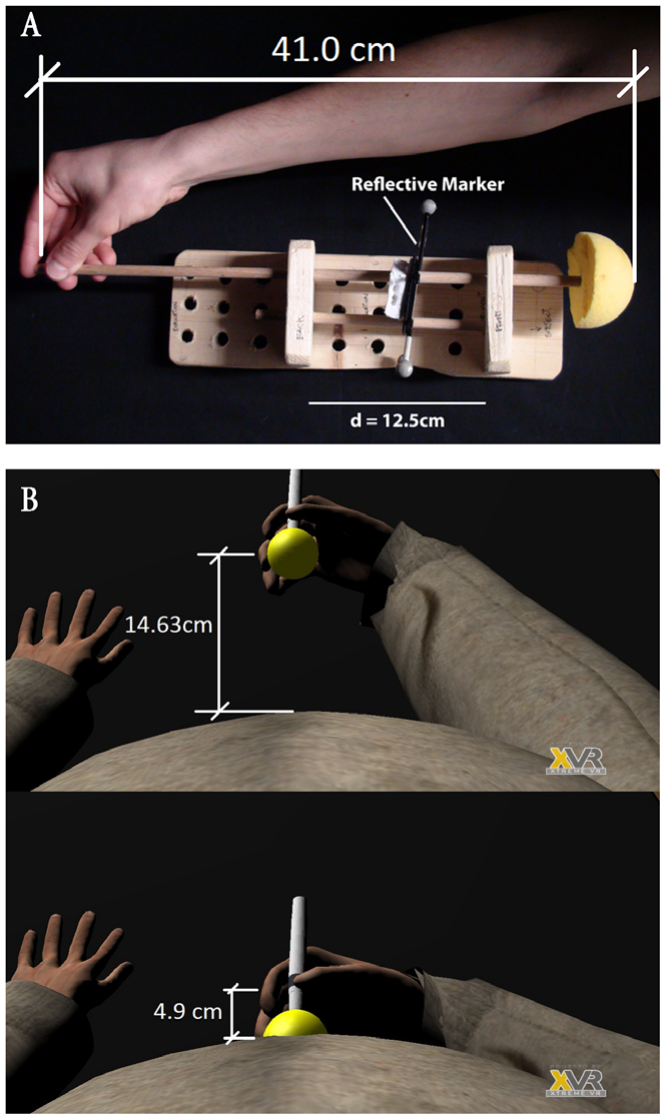
The rod used for modifying belly size during evaluations and tapping during the experiment. (A) The physical rod - note the reflective marker used for optical tracking of the rod movements. (B) The virtual rod.

**Figure 4:**
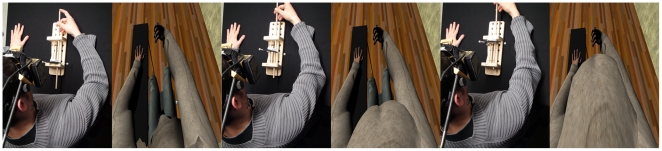
Modifying the size of the virtual belly with the rod. The closer the rod to the participant (from left to right) the smaller the size of the virtual belly. The yellow pad at the end of the rod was removed for the purposes of measurement.

There was a rod on the table in front of them (Figure 3) that was not visible due to the HMD. The purpose of the rod was twofold. First it was designed as a measuring device for a body size change measurement during evaluation phases, and second as a haptic device for belly prodding during the experiment. The participants were guided by the experimenter to hold one end of the rod. They learned that as they moved the rod forward and backwards the belly size of their virtual body would change, grow larger or smaller associated with the rod movements (Figure 4). They were told to adjust the virtual belly size until they perceived it to be the size of their own real belly. Once the participants were satisfied with the size they indicated this to the experimenter, and a corresponding measurement henceforth referred to as the initial size *Sbefore*, was recorded. This measurement corresponded to the self-estimated absolute size of the belly, from spine to belly button and in centimeters. It is important to note that during this procedure the rod device was positioned to ensure that the end nearest the participant could never actually touch their real belly, so that the only clue to size was visual.

Then after a period of a few seconds of dark screen display, the main experimental phase started, where the participant was asked to repeatedly push the rod towards his belly area (now adjusted so that the device could strike the participant).

**Figure 5:**
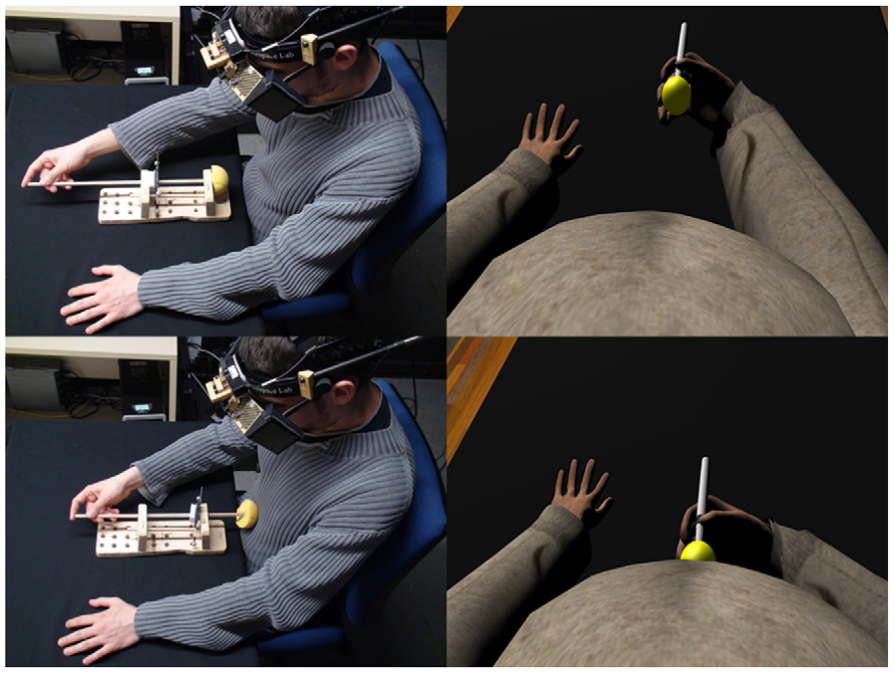
Synchronous condition. Whenever the participant pokes his belly with the rod, the virtual belly is touched at the same time by the virtual rod.

During this time the physical rod device was registered with a virtual rod device visible in the virtual display. Whenever the participant poked his virtual belly in the synchronous condition, the device poked their real one in order to provide synchronous visual-tactile stimuli (Figure 5). This way, in the synchronous mode, the participant could see the virtual belly being tapped while feeling his real one being tapped. In the asynchronous condition the movements of the virtual rod were unrelated to the participant's movements of the real rod. Instead, we used pre-recorded movements for the virtual rod in order to break the visual-tactile coherency of the participant's movements and the tapping sensation on his belly. The same sequence of prerecorded movements was used for all participants.

The participant was asked to always look at his virtual belly during the whole experiment. Throughout this part of the experiment, the participant heard music with a complex irregular rhythm through a pair of headphones. The participant had been told that his task was to try to follow the rhythm of the music while moving the rod. This continued for 4 minutes. Once the 4 minutes were over, the subject was shown a short period of black display and was asked to manipulate the rod as before, in order to indicate the felt size of his belly.

Immediately after this the experimenter asked the participant whether “your body size is different from how it normally is?” and a ‘yes’/‘no’ answer was required. If the answer was ‘yes’ then they were asked “How has it changed?” which could be answered freely. We will refer to this as the *immediate question*.

The HMD was then removed and the participant was asked to complete a post-experimental questionnaire. They then repeated all of the above for a second time, but now with the second condition (for example, asynchronous if the first had been synchronous).

### Response Variables

The first set of response variables was based on a questionnaire (apart from the immediate question). This consisted of 11 statements, each responded to on a 0 to 10 scale, where 0 indicated complete disagreement, and 10 complete agreement with the statement made.

- Q1. It seemed as if I was feeling the touch at the location of the yellow ball.
- Q2. It seemed as though the touch I felt was caused by the yellow ball touching the virtual body.
- Q3. I felt as if the virtual body were my body.
- Q4. It seemed as if the touch I was feeling was located somewhere between my felt body and the seen body.
- Q5. At some point during the experiment I felt my body expanding to take on the shape that I saw.
- Q6. At some point during the experiment I felt heavier than usual.
- Q7. I was aware of a conflict between my felt body and the seen body.
- Q8. It seemed as if I had more than one body.
- Q9. After taking off the head-mounted display I felt the need to check that my body size was really smaller than the virtual body I had seen.
- Q10. I felt an after-effect as if my body had become swollen.
- Q11. The illusion of having a swollen body was very strong during the experience.

Q1–Q3 are based on the questionnaire in [1] but adapted for this situation. Q4 and Q8 were considered as control questions, i.e., statements that were not expected to be generated by the illusion, similar to two of those used in [1]. The remaining questions (Q5–Q7, Q9–Q11) were new and exploratory. We expected Q5 and Q6 to indicate the illusion, Q9 and Q10 were to examine whether the illusion vanished after completion of the experiment, and Q11 was an overall summary indicator of the strength of the illusion, and quite a demanding question since it refers to having a ‘very strong’ illusion. We did not have preconceptions about Q7.

Based on previous work we therefore expected Q1–Q3 to have greater scores indicating the illusion in the synchronous condition compared to the asynchronous, but no significant difference for questions Q4 and Q8.

The second response variable was size change (*ΔSize*) where *ΔSize* = *Safter*−*Sbefore*. We expected this to be significantly higher on the average in the synchronous compared to the asynchronous condition. In other words people would tend to overestimate the body size in the synchronous condition, if that condition had led to an ownership illusion.

The third indicator of the illusion would be a correlation between *ΔSize* and the illusion-related questions. The greater the subjective sense of the illusion the greater the expected size of the drift on average.

### Questionnaire Results

**Figure 6:**
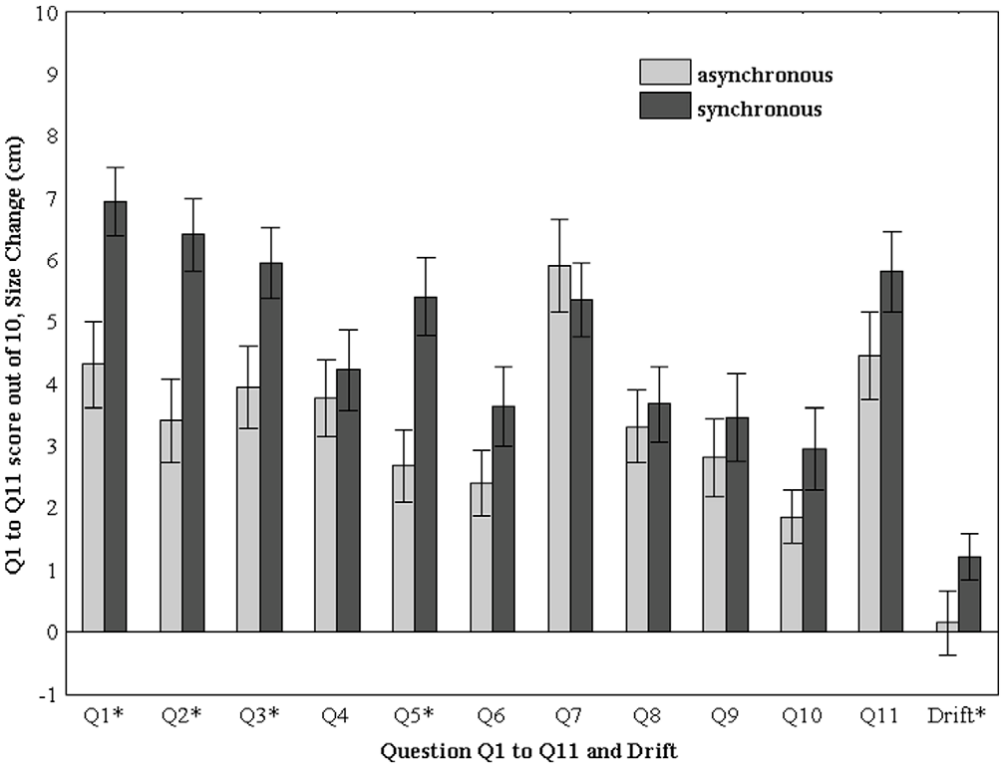
The means and standard errors of the questionnaire responses Q1 to Q11, and of the *ΔSize* measurement.

Figure 6 shows the means and standard errors of the questionnaire responses. Paired non-parametric sign tests show significant differences for the illusion questions (Q1–Q3) and no significant difference for the control questions (Q4 and Q8). Table 1 gives the medians, median deviations and significance levels for the complete set of questions.

**Table 1:** Median (M) and Median Deviation (MD) for the Response Variables, and P the two-sided significance level for the paired sign tests of difference between medians.

	Asynchronous		Synchronous		
Var	M	MD	M	MD	P
**Q1**	**4.0**	2.5	**7.0**	1.5	**0.001**
**Q2**	**3.0**	3.0	**7.0**	2.0	**0.012**
**Q3**	**3.0**	2.0	**6.0**	1.0	**0.041**
**Q4**	**3.0**	2.0	**4.5**	2.5	1.000
**Q5**	**2.0**	2.0	**6.0**	2.0	**0.002**
**Q6**	**2.0**	2.0	**3.0**	2.5	0.119
**Q7**	**7.0**	1.5	**6.0**	3.0	0.824
**Q8**	**3.0**	2.5	**3.0**	3.0	1.000
**Q9**	**2.0**	2.0	**3.0**	3.0	0.582
**Q10**	**1.0**	1.0	**2.0**	2.0	0.109
**Q11**	**4.5**	3.0	**7.0**	2.0	0.118
***ΔSize***	**0.31**	1.03	**1.78**	1.43	**0.052**

For Q1 to Q11 the scores are out of 10 and *ΔSize* is in cm.

In addition to the main questions Q1–Q3, Q5 lends strong support to the illusion occurring more in the synchronous than in the asynchronous condition, but Q6 indicates that the feeling of body size expansion was not associated with a feeling of being heavier. Q9–Q10 indicate that the illusion ended after the experimental trial with no difference between synchronous and asynchronous conditions. Q7 shows no difference between the asynchronous and synchronous conditions, but the two median scores are quite high, indicating a possible conflict between the seen and own body. Q11 shows a greater median score for the synchronous condition but is not significantly higher than the asynchronous.

Regarding the immediate question 12/22 in the synchronous condition answered affirmatively that their body size was different, compared with 5/22 in the asynchronous condition (P = 0.030). Almost all subjects indicated that the difference was due to their body size feeling ‘slightly bigger’ or ‘bigger’ than normal.

### Size Change Results

The final row of Table 1 shows the results for *ΔSize*, that is the difference between the after and before measures. The result indicates greater *ΔSize* in the synchronous condition compared to the asynchronous. Moreover, *ΔSize* was greater for the synchronous condition compared to asynchronous for 16 out of the 22 participants. The null hypothesis of equal probability of *ΔSize* in the synchronous condition being greater or smaller than in the asynchronous is rejected with P = 0.026 using the binomial distribution - this significance level being the probability of observing 16 or more cases of *ΔSize* being greater in the synchronous condition. See also ‘Methods’ for a further discussion of the comparison of *ΔSize* between the two conditions.

**Figure 7:**
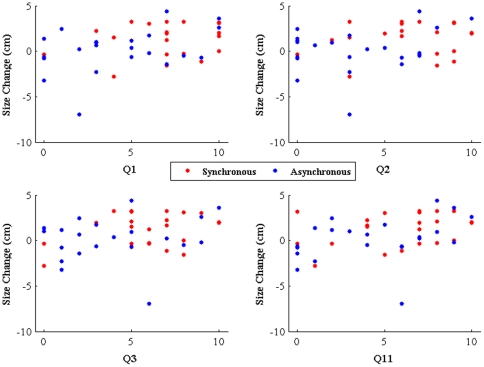
Scatter diagrams showing the relationship between *ΔSize* and the scores on Q1–Q3 and Q11, by condition (synchronous and asynchronous).

The most interesting evidence in favor of the illusion is that there are positive correlations between the questionnaire scores indicating the illusion and *ΔSize*. Figure 7 shows plots of *ΔSize* for Q1–Q3 and Q11 by condition. These indicate that there was a positive association between the degree of illusion as recorded by the questionnaire responses and the size of *ΔSize*, but independently of condition. Table 2 shows the Spearman rank correlation coefficients and the corresponding significance levels for all of the questions. As expected the three questions Q1–Q3 indicating the illusion are each significant (just outside the conventional limit for Q2). However, one of the control questions (Q4) is also significant. Q7–Q10 are not significant. The greatest correlation is between Q11 (the overall strong statement of the illusion) and *ΔSize*.

**Table 2:** Spearman's Rank Correlation Between the questionnaire results and *ΔSize*.

	r	P
Q1	0.35	**0.020**
Q2	0.29	**0.053**
Q3	0.32	**0.036**
Q4	0.34	**0.025**
Q5	0.35	**0.022**
Q6	0.31	**0.038**
Q7	0.13	0.403
Q8	−0.13	0.391
Q9	0.14	0.374
Q10	0.27	0.074
Q11	0.45	**0.002**

## Discussion

This experiment provides evidence supporting the notion that a body size illusion can be produced in an immersive virtual environment where participants see a virtual body substituting their own body from a first person perspective. There were various reinforcements that may have contributed to this illusion: First, there was first person perspective, used previously in [6] together with synchronous visual-haptic body tapping, and in [12] where it was shown to be the strongest out of three factors (perspective position, visual-tactile synchrony, and visual-motor synchrony with respect to head movements seen in a virtual mirror). Second, there was proprioceptive support for the illusion since the participant's arm was stretched out beyond the real body size, and the apparent distance between the participant's hand and virtual belly was only about 5cm – the distance from the hand to the end of the virtual rod - at the moment of touch (Figure 3B). Additionally always touch was felt on the real body and synchronized with the real hand movements (since the touch was self administered). However, in one condition the movements were synchronous with the movements of the virtual hand and rod, and therefore there was visual-tactile and visual-motor synchrony, and in the other condition there was visual-tactile and visual-motor asynchrony since the virtual hand movements were from a pre-recorded animation.

**Figure 8:**
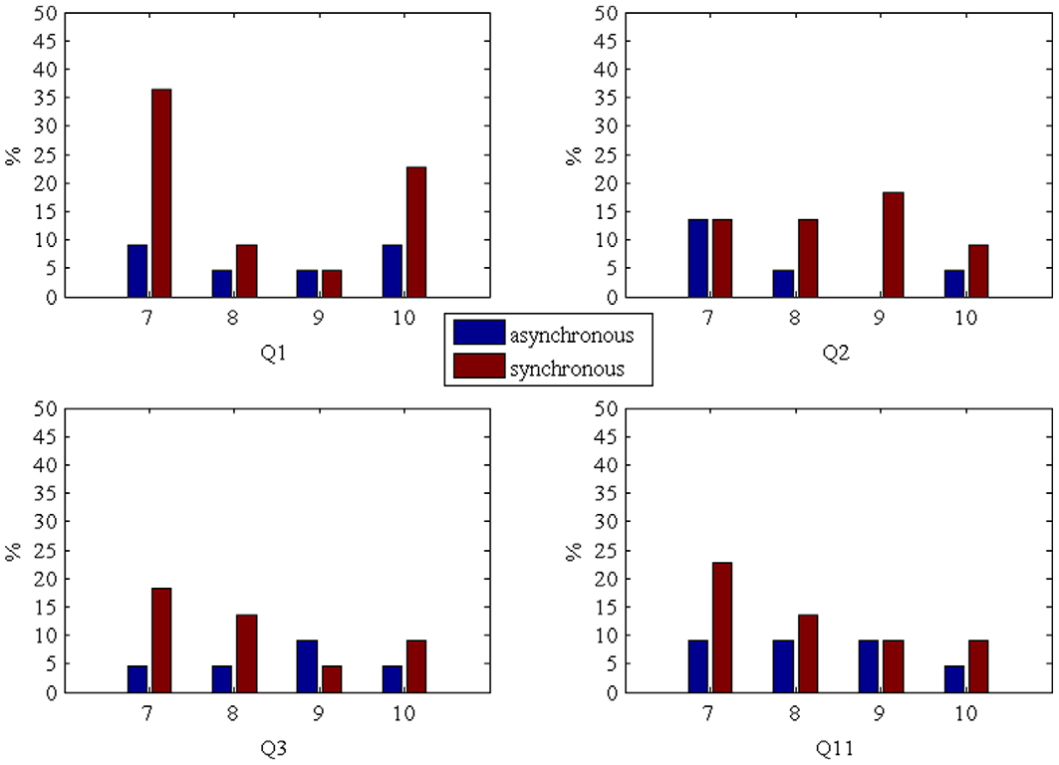
Bar charts for the distribution of questionnaire scores of 7 or more for questions Q1–Q3 and Q11.

However, even in the asynchronous condition the illusion occurred for some participants. For example, Figure 8 shows bar charts for Q1–Q4 and Q11, for scores of 7 or more out of the maximum score of 10. It reveals that in the asynchronous condition the proportions of participants who reported these relatively high scores were 27%, 23%, 23% and 32% respectively for Q1–Q3 and Q4 (the equivalent proportions for the synchronous condition were 73%, 55%, 45% and 55%).

Since we had asked participants to concentrate visual attention only on the yellow ball striking their virtual body, the experiment was similar to [6] where the head was fixed looking down at the body towards the point of tactile stimulation. As was the case with that experiment in the absence of free head movement additional synchronous stimulation was necessary to improve the chance of generating the illusion. It should be noted, however, that the synchronous condition was more than just synchronous visual-tactile – it also included some visual-motor synchrony, since the virtual hand moved in correlation with the real hand (and as reported in Methods the latency was very low). Additionally therefore the asynchronous condition was also asynchronous with respect to visual-motor as well as visual-tactile. Moreover, since a pre-recorded animation was used in this condition, it is possible that participants may have been at least subliminally aware that the hand movements were not movements that were recognizably their own. However, evidence suggests that people tend to misattribute the hand of an experimenter to be their own even when the hand deviates in its movements from their own until the discrepancy between the two becomes large [17], [18], [19]. In the current experiment in the asynchronous condition participants were performing the same type of movement as the animated virtual hand – that is, making small irregular prodding motions as per instructions in time with the music, while the virtual hand animation had been prerecorded by an experimenter who was not listening to the music at the time of the recording. It is possible that there was some degree of misattribution and also that the participants might have been influenced to copy the virtual hand movements, so there could have been more synchrony in the asynchronous condition than desirable. Unfortunately, we do not have the participants' real hand movements recorded so that we cannot test this. Having said all this, of course we did, as reported, find significant differences in subjective and measured responses between the asynchronous and synchronous conditions, but we cannot disambiguate the possible different effects of the visual-tactile and visual motor factors.

The evidence suggests that *ΔSize* was generally higher in the synchronous condition than in the asynchronous although the magnitude of *ΔSize* is quite small (the median is 1.78cm and the mean is 1.22cm in the synchronous condition compared to the overall rod length of 41cm). However, it is interesting to observe that synchronous multi-sensory bottom-up information was sufficient to slightly shift perceptual judgments of belly size towards the virtual belly, partially overriding the top-down knowledge of true belly size. Also, the size judgments were made several seconds after the end of the stimulation (allowing time for the experimenters to reposition the rod for the measurement, and also for the participants to move the rod in making their size judgment). Therefore the final measurement does not allow for any possible decay in the illusion of greater size over time. It would be useful in further work on body ownership illusions to try to estimate the temporal course of such displacement measures. Proprioceptive drift in the context of the rubber hand illusion is similarly not large – between 15–30% of actual distance between the fake and real hand [8], [20], [21], also perhaps because the time to actually take the size measurements, and the actions involved in doing so, already diminish this aspect of the illusion being measured.

As well as being higher on the average for the synchronous compared to asynchronous condition *ΔSize* was significantly correlated with the questionnaire scores. Since each type of measurement was realized by quite different methods the fact that they correlate is an important sign of the occurrence of the illusion. Note that *ΔSize* correlated with questions indicating that the felt touch was coming from the enlarged belly (Q1, Q2), ownership of the virtual body (Q3) and questions relating to body size and weight (Q5,Q6). The only anomaly was that it also correlated with the control Q4, indicating that the touch was felt somewhere between the seen and felt body. On this latter point the scores for Q4 although low, and not significantly different between the synchronous and asynchronous conditions, are highly positively correlated with Q1–Q3 and Q11 (all P<0.01) which is accounted for by high correlations between these questions in the asynchronous condition. This correlation between the control and the illusion questions has been noted before in [9] where it was concluded that participants respond to the questionnaire as a whole, and that the differentiation between illusion-related and control questions may not be the best approach.

In the context of the rubber hand illusion it has been argued that the ownership illusion is dissociable from proprioceptive drift although they may be correlated in practice [22], [23]. However, in the case of this body size experiment, the size estimates are not like proprioceptive drift where the position in space of a limb is mislocalized. In the body case the participants directly manipulate their virtual body size seen from the first person perspective making a judgment about when it is apparently equal to their real body size. During this evaluation they move the rod with their unseen hand, and the only visual feedback is the corresponding changing size of the virtual belly.

The question of the number of body representations in the brain and the nature of those representations [24], [25] and their susceptibility to the rubber hand illusion [26], [27] is an important topic in understanding how the brain represents the body and the source and limitations of each type of body representation. The current experiment shows that the illusion of ownership of a distorted body is possible, and previously it has been shown that ownership of a different body can be achieved including gender changes [6], [12]. Here we have added to that by showing a body size change is possible. In the way that this was produced with multisensory correlations (visual, haptic, proprioceptive) and the way that it was assessed (subjective and perceptual size judgments) it is not possible to say that this illusion belongs exclusively to the ‘body image’ (perceptual judgments) or ‘body schema’ (action, proprioception) domain. A further experiment would be necessary that separates out the different combinations (visual perspective, movement, haptics) and assesses their independent effects.

A final point is that in this experiment the visual-tactile stimulation was self-administered. It would be interesting to know how the results would change if the stimulation were not self-administered but performed by another agency (the experimenter or through an automatic method). In this case the visual-motor synchrony would be removed from the setup, but could be reintroduced through other means (e.g., body and limb movements unrelated to the prodding action). In this way also the separate effects of visual-tactile and visual-motor synchrony could be assessed. The rubber hand illusion relies on irregular tactile stimulation, so that participants cannot predict when the next stroke will be. It is thought that such unpredictability is an important factor in a Bayesian model for the occurrence of the illusion, since the coincidence of the visual and tactile stimulation is a more unlikely event than were the stimuli to be highly regular. Therefore this leads to large increase in the probability that the rubber arm is the arm of the subject. For example Armel and Ramachandran [7] observed that ‘Subjects reported that the more random and unpredictable the touch (if synchronized), the more vivid the illusion’. In the case of our experiment since the stimulation is self-administered it cannot be unpredictable (though it can be irregular). In fact it appears that neither self-stimulation nor predictable tactile stimulation have ever been systematically explored with respect to the RHI, and perhaps the assumption that unpredictability and irregularity are necessary needs to be examined empirically. Of course there are many other aspects of the delivery of the visual-tactile stimulation that need to be considered (position, strength, aspects of quality, and so on) but the point about predictability is one that is generally believed and widely adhered to.

Virtual reality is typically thought of as a way to place people in a simulated world - in other words to manipulate their sense of place or presence [28]. Here we have shown that virtual reality, with appropriate synchronous multisensory stimulation, has a much greater transformational capability that has not hitherto been exploited - the capability to transform not just place but also the self. Although similar results to the present ones have been obtained with a much simpler setup, using a manikin and video streamed through head-mounted displays [6], the potential for real-time virtual environments in this context is much greater. VR includes the ability to have many different forms of representation, arbitrary and dynamic changes to the scenario including the body, with complete control over what is displayed, with also the easy ability to connect together, or separate, different multisensory data streams.

There are interesting applications for this result. The first is entertainment, where participants in an immersive real-time virtual reality could experience what it is like to have certain bodily features that they do not have in reality. However, this also could have important implications for allowing a person to change perspective on the world. Having the illusion that our body is different (a man becomes a woman or vice versa, a thin man becomes fat - or vice versa) applied in the context of social virtual environments could become a powerful method for enhancing understanding, empathy and even personal transformation.

If it is the case that it is possible to generate the illusion that a thin person is fat, then it should be possible to generate the converse illusion that a fat person is thin. This could have implications for use in a goal-directed therapy, where patients could, from a first person perspective, experience themselves as being how they want to be as a strong motivator for successful completion of the therapy program. In [29] it was shown that when a subject sees an avatar representation of him- or herself, from a third person perspective, eating healthily with positive consequences for virtual body appearance, that this has a positive effect on subsequent behavior in reality. We speculate that this type of effect could be made significantly more powerful when experienced from a first person perspective in an immersive environment with a body ownership illusion. There is a body of work that uses virtual reality in the treatment of body size distortions (see [30] for a review), but to our knowledge have never used the type of setup described here - first person perspective view of a collocated virtual body via a head-tracked stereo wide field-of-view head-mounted display, and multisensory stimulation. Moreover the issue of body ownership has never been addressed in that field of research which has mainly concentrated on therapeutic outcome.

## Materials and Methods

### Ethics

The experiment was approved by the Comité Ético de Investigación del IDIBAPS (Hospital Clínic, University of Barcelona) and written informed consent was obtained from all participants.

### Equipment

The stereoscopic head-mounted display was a Fakespace Wide5, which has a field of view of 150°×88° with an estimated 1600×1200 resolution displayed at 60Hz, and the head-tracking was with an 6-DOF Intersense IS-900 device.

### Rod Calibration

Tracking of the rod was performed by an Optitrack optical infrared system which tracked reflective markers attached to the rod. A calibration of the real rod was performed in order to ensure a correspondence of the virtual and the real rod's movements. This was achieved by recording two 3D points *c* and *f* being respectively the closest and furthest rod positions from the real belly of the participant (Figure 4).

The coordinates *c* and *f* were then mapped to the smallest and largest sizes of the virtual belly. A linear interpolation was used to determine the size of the virtual belly for each position of the real rod between *c* and *f*. The closest position of the rod was mapped to the minimum belly size of the avatar and the furthest to the maximum size. A small random offset (between −0.5 and 0.5 cm) was added to pre-defined minimum (14.8cm) and maximum (47.3cm) measures of the belly. Those minimum and maximum values were chosen to be exaggerated in order to offer the participant a large range of possible belly sizes. Sizes were measured from the spine to the belly button of the avatar and the initial position of the rod was randomized at each evaluation. The reason for adding random values to the extrema was to make it difficult for participants to remember the position of the rod when making the initial estimate of body size compared to the final measurement, and also across the two trials.

By moving the real rod forward and backward, the participant was able to modify the size of the virtual belly during the measurement phases. Once the participant signaled that the size of the virtual belly corresponded to the size of his own belly this value was recorded (Figure 4).

The *Sbefore* value was determined by the above measurement protocol which was performed before the start of the main experimental phase. This was the first task the participant had to perform after familiarizing himself with the virtual environment and prior to the period of stimulation. The *Safter* measurement was recorded using the same protocol, but performed right after the end of the stimulation phase.

### The Models

The environment was modeled in 3DStudio Max and rendered using the XVR system [31]. Animation and rendering of the avatar was performed with the HALCA library [32].

We modified the avatar's belly by using 2 morph targets, which were interpolated in a GLSL shader to visualize the appropriate size. For the experiment this size was exaggerated (45 centimeters between the spine of the avatar and his belly button) in order to ensure a large difference between the size of the subject's real belly and the virtual one.

In order to map the movements of the participant's arm to those of the avatar's arm we used a morph animation as follows. We modeled an animation that moves the right arm of the avatar from touching the belly to an extended position (as shown in Figure 5). In HALCA the extended position of the tracked rod was associated with the end of the animation, and the close position of the rod with the start of the animation. Positions in between were mapped to interpolated key frames between the start and end of the animation.

### Synchrony and Asynchrony

In the synchronous condition, the movements of the reflective marker attached to the rod were read in each render cycle, in order to map the backward and forward movements of the rod to the movements of the right arm of the avatar holding the virtual rod. Once the rod was positioned and the optical marker calibrated, whenever the participant touched his belly with the rod, the virtual rod touched the belly of the avatar, providing synchronous visual-tactile stimulation.

For the asynchronous mode, our initial solution had been to simply add some delay to the movements of the virtual rod. This solution was not suitable for the purpose of the experiment due to the relative high frequency of belly poking. Since our goal was to break the visual-tactile coherence (i.e. the participant should not see the rod touching the virtual belly when the real rod touched his real belly) we could not use this solution based on delays. In order to overcome this problem we pre-recorded rod movements and these were used as playback during the asynchronous condition.

### Latency

The Optitrack V100 (Natural Point) cameras that were used for the optical tracking of the rod have a frame rate of 100Hz and an estimated latency (provided by the manufacturer Natural Point) of 10ms. There remains the processing of the image and the transfer over the USB. Natural Point estimates 15–16ms to be a good upper bound for the whole processing of the information from the cameras to the network (network latency was negligible since we were using a local network with a ping value <1ms. Based on the refresh rate of the HMD, the fairly simple virtual environment (composed of approximately 30,000 polygons) was displayed at 60 frames per second. Hence the display function was called every 16.7ms. As a consequence, the delay between getting the movements of the rod and displaying these in virtual reality was <1ms in the synchronous condition.

### Statistical Analysis

Standard ANOVA should not strictly be used on questionnaire data since the responses are on an ordinal scale. Nevertheless this is frequently done, and for comparison purposes the results of a one-way ANOVA for the within-groups repeated measures design is given in Table 3. There was no effect of group (SA or AS), in other words no order effect, for any question (the lowest P = 0.12 on Q8). The results correspond to those of Table 1.

**Table 3:** Mean and Standard Deviation of Questionnaire Scores by Group and Condition.

	SA				AS				
	Asynchronous		Synchronous		Asynchronous		Synchronous		
	Mean	SD	Mean	SD	Mean	SD	Mean	SD	P
**Q1**	**4.0**	3.5	**7.1**	2.9	**4.7**	3.1	**6.8**	2.3	0.001
**Q2**	**3.3**	3.6	**6.5**	2.9	**3.5**	2.7	**6.3**	2.8	0.001
**Q3**	**3.6**	3.2	**5.8**	2.7	**4.4**	3.0	**6.2**	2.7	0.007
**Q4**	**3.4**	3.1	**4.8**	3.4	**4.2**	2.7	**3.6**	2.6	0.472
**Q5**	**2.6**	2.6	**4.9**	3.4	**2.8**	3.1	**6.0**	2.3	0.000
**Q6**	**2.3**	2.6	**4.1**	3.2	**2.6**	2.5	**3.1**	2.7	0.027
**Q7**	**5.2**	3.8	**5.1**	2.8	**6.8**	3.1	**5.7**	2.9	0.441
**Q8**	**3.0**	2.6	**2.5**	2.4	**3.7**	3.0	**5.1**	2.8	0.529
**Q9**	**2.0**	2.9	**3.3**	3.5	**3.8**	2.9	**3.7**	3.3	0.372
**Q10**	**2.0**	2.3	**2.3**	2.9	**1.7**	1.8	**3.8**	3.2	0.013
**Q11**	**3.7**	3.3	**5.4**	3.0	**5.4**	3.3	**6.3**	3.1	0.044
***ΔSize***	**0.7**	2.1	**1.5**	1.6	**−0.5**	2.7	**0.9**	2.0	0.119

SA is the group that first received the Synchronous condition and then Asynchronous, and vice versa for AS. P is the significance level for the test of equality of means in the within-group repeated measures one way ANOVA.

The result for *ΔSize* is an exception. However, in this case the results are heavily influenced by the trial number. If we consider only the between-groups experiment consisting of each participant's first trial, then *ΔSize* is significantly higher for the synchronous condition (P = 0.046). However, here the ANOVA does not satisfy the requirement for normality of the residual errors of the model, using a Jarque-Bera test (P<0.01) [33]. A Box-Cox transformation [34] was found which resulted in normally distributed residual errors, and in this case the significance level for the main effect becomes P = 0.038.

Finally we can consider *ΔSize* in the synchronous and asynchronous conditions separately and use t-tests for the hypothesis that the means of *ΔSize* are zero in each case. In the two conditions the means and standard errors are 1.22±0.38cm and 0.15±0.51cm respectively. Using Jarque-Bera tests *ΔSize* is compatible with a normal distribution in the synchronous case (P = 0.18) but not in the asynchronous case (P = 0.02). However, the latter is due to one outlier, and if this is eliminated then the asynchronous *ΔSize* is also compatible with normality. Then the hypothesis of zero mean is rejected for the synchronous case (P = 0.008) and not rejected for the asynchronous case (P = 0.24 with the outlier removed, and P = 0.78 including the outlier).

## Supporting Information

Movie S1 Movie showing all of the major phases of the experiment including the sound track that was used.
(M4V)
